# Statistical modeling and estimating number of healthy life years lost and healthy life expectancy in India, 2000–2019

**DOI:** 10.1002/agm2.12269

**Published:** 2023-09-27

**Authors:** Diptismita Jena, Prafulla Kumar Swain, Manas Ranjan Tripathy, Pravat Kumar Sarangi

**Affiliations:** ^1^ Department of Statistics Ravenshaw University Cuttack Odisha India; ^2^ Department of Statistics Utkal University Bhubaneswar Odisha India

**Keywords:** healthy life expectancy (HLE), healthy life year lost (HLYL), life expectancy (LE), mortality, statistical modeling

## Abstract

**Objective:**

In this study, our objective is to propose various models to estimate healthy life year lost (HLYL) and healthy life expectancy (HLE) in India.

**Methods:**

The HLYL and HLE were estimated and further these estimates were compared with the direct life table method and the World Health Organization (WHO) method. From the mortality perspective, we have developed a log‐logistic model for estimating the parameter (bx), which is characterized by HLYL. The results were compared with other models, such as the Gompertz and Weibull model. Here, we have also obtained the HLE by subtracting HLYL from the total life expectancy.

**Results:**

The result shows an increasing trend of HLYL among the male, female, and the total population in India.

**Conclusion:**

From the log‐logistic model, the HLYL was estimated as 8.79 years, 8.36 years, and 9.38 years for the total, male, and female populations, respectively, in India during 2019.

## Graphical abstract

Healthy Life Expectancy (HLE) is an important indicator of mortality and morbidity. HLE can be obtained by subtracting number of Healthy Life Years Lost (HLYL) from the Life Expectancy (LE). In this study we have computed HLE and HLYL for Indian population using Log‐logistic model and compared the results with Gompertz and Weibull models.

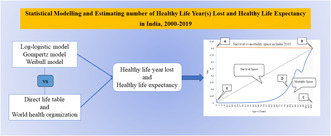

## 1 INTRODUCTION

From the last 2 centuries, the Global burden of disease (GBD) has been widely studied in the field of epidemiology.^1^ GBD is the most comprehensive local, regional, and global research program to quantify the magnitude of health loss in terms of mortality and morbidity due to several diseases, injuries, and risk factors for specific time points. The scientific and systematic study of GBD enables us in understanding the rapidly changing health challenges and difficulties faced by people across the world. Life expectancy (LE), healthy life expectancies (HLEs), and disability adjusted life years (DALYs) are the basic components of the study of GBDs.^2^ These health components provide important information on potential sociodemographic factors pertaining to longevity figures and their transition, as well as global perspectives for reaching the sustainable development goal (SDG‐3) of ensuring healthy lives and promote well‐being for all at all ages.^3^

According to the World Health Organization (WHO), the term health is defined as a state of complete physical, mental, and social well‐being and not merely the absence of diseases or infirmity.^4^ Health can be considered as a stochastic variable because it is associated with uncertainties due to sudden changes in the state of illness, diseases, and accidents.

LE generally depends upon the expected number of years lived and is concerned with the health status of an individual. LE is the average number of years expected to be lived at a particular age, considering current mortality conditions. Therefore, HLE measures the number of years that a population may anticipate to live in good health assuming that they survive the current state of health and mortality circumstances. For instance, if a person has an LE of 80 years and a health adjusted life expectancy (HALE) of 74 years, 6 of the 80 years are effectively “lost” due to ill health. Major planning in health care is based upon objective indicators, such as mortality, morbidity, or disability statistics. The combination of mortality figures with LE, HALE and healthy life years lost (HLYL) provides meaningful health outcomes at the population level.

Over the years, many general health indicators have been proposed, however, relatively few have been applied. The WHO provides the most powerful health estimate as HALE. The concept of HLE was introduced in the 1960s and was developed by Sullivan.^2^ LE is an indicator of mortality in a population, whereas HLE is an indicator of both mortality and morbidity. A demographic transition from high to low levels of mortality and fertility, along with a rise in LE has resulted in population aging.^5^, ^6^, ^7^

The health transition of India began at a low life expectancy at birth (LEB; ie, 24.8 years).^8^ In the early 1980s, female LE exceeded male LE at birth.^9^ Gradually, the overall life expectancy in India increased and has been recorded as 70.79 years, 69.52 years, and 72.17 years for the total population, male population, and female population, respectively. HLE was recorded as 60.3, 60.3, and 60.4 years for total, male, and female populations,respectively.^3^ Based on the latest Sample Registration System (SRS) abridge life table report (2015–2019), the LE for the urban and rural populations in India was recorded as 73 years and 68.3 years, respectively.^10^ The average LE of the global population in the year 2019 was 73.3 years.^11^ It is widely known from previous reports that LE at birth in India has doubled over the past 50 years. Although this accomplishment is commendable, it is important to recognize that these additional years might not necessarily be years of good health. India continues to face challenges with infectious diseases, malnutrition, and the rapid rise of noncommunicable diseases, as well as age‐related changes in physical health that lead to disability.

The aging process is a significant factor contributing to the increasing burden on social, economic, and health care systems in virtually all countries. Developing nations, in particular, experience the dual challenge of dealing with illnesses and disabilities, which poses a threat to the overall quality of life for their aging populations. With India being the most populous country in the world and experiencing a rapid growth in its older population, there is a pressing need to estimate healthy LE.

HLE can be obtained by subtracting the number of HLYL from the total LE, where HLYL is the number of years that a population generally loses due to their illness by assuming they survive at a current state of health and mortality conditions. Therefore, HLYL plays a major role to understand the severity of public health issues and helps to allocate scarce resources effectively in public health planning to set priorities for prevention, and to control the GBD among the populations.

To measure the health state of a population, HALE plays an important role and was provided by the WHO. The practical approach for estimating the HLE was given by Jagger et al.,^12^ Romero et al.,^13^ estimated the HLE in Brazil by applying the Sullivan method. The estimation of LE and HLE for the Japanese population was done by Tokudome et al.,^14^ The interconnection between the HLYL and the Weibull shape parameter is observed by Matsushita et al.,^15^ and further explored by Skiadas and Skiadas.^16^ Skiadas et al.,^17^ estimates the LE and HLE for men and women in France during 1900 to 2017 and forecasted the same for the year 2060. Skiadas and Skiadas,^18^ have also estimated the HLE and HALE using best fit logistic model during 1751–2016 in Sweden and found very close estimates as given by the WHO.

It is crucial to monitor the change in levels of physical and mental wellbeing in a population, as increased longevity alone holds little value without a healthy life. The data on LE and HLE allows us to recognize disparities in overall health among different groups according to age, gender, socioeconomic status, living conditions, and other factors. This information also enables us to identify and measure the impact of illness on the overall health of the population. The information on LE, HLE, and HLYL is useful in determining the allocation of resources for health promotion and in providing an improved understanding of the determinants of health. This information can be used to predict the future needs of a population, to provide information in planning of health and social services, and to identify trends and inequalities present in the population. The aim of this research is to calculate HLE and HLYL for the Indian population using the log‐logistic model and also to compare the results with Gompertz and Weibull models.

## 2 MATERIALS AND METHODS

### 2.1 Data sources

The secondary data have been collected on various columns of complete life table from 2000 to 2019 for India from http://www.mortalitytrends.org. The information on various components of complete life table, that is, $d_{x}$(number of deaths at age *x*), $m_{x}$(age specific mortality rate), $l_{x}$(age specific population), and $e_{x}$ (life expectancy) were collected and combined together. By using this information, we have estimated HLYL and LE for the Indian population for the years 2000–2019. Data on LE and HLE were collected from the WHO website on “Life expectancy and Healthy life expectancy Data by country” for the years 2000, 2010, 2015, and 2019 for the comparison purpose.

### 2.2 Methodology

In this study, we have applied four different methods to estimate HLE and HLYL for the Indian population and further compared with the values provided by the WHO. The four different methods are (a) the direct method, (b) the Gompertz model, (c) the Weibull model, and (d) the log‐logistic model. The detailed methodologies for estimating HLE and HLYL have been discussed below.

#### 2.2.1 Direct method

The direct methods (without using a model) for estimating HLYL were given by Skiadas and Skiadas,^16^, ^19^, ^20^ which is based on averaging the health state of a population. The use of life table data for this purpose is advantageous because it can be applied to any population, regardless of whether or not direct data on health and diseases are available. The full life table from the Human Mortality Database (HMD) is followed by four more columns for the estimation of bx. In the first, the cumulative mortality is estimated from $M_{x}=\sum_{0}^{x}m_{x}$. The average mortality $\frac{M_{x}}{x}=\frac{\sum_{0}^{x}m_{x}}{x}$ is provided in the next column whereas the Person Life Years Lost $(\text{PLYL})=\frac{{\mathrm{xd}}_{x}}{\sum_{0}^{x}m_{x}}$ are calculated in the next column. For this very important information, an interesting graph is provided. The graph follows a growth process until a high level and a decline in the remaining lifespan period. It the next column, the HLYL estimator $b_{x}$ is provided by dividing the PLYL by the $l_{x}$ from the life table.

A graphical approach for this methodology is shown in the following figure of mortality spaces where both mortality and survival are presented by the corresponding areas.

**FIGURE 1:**
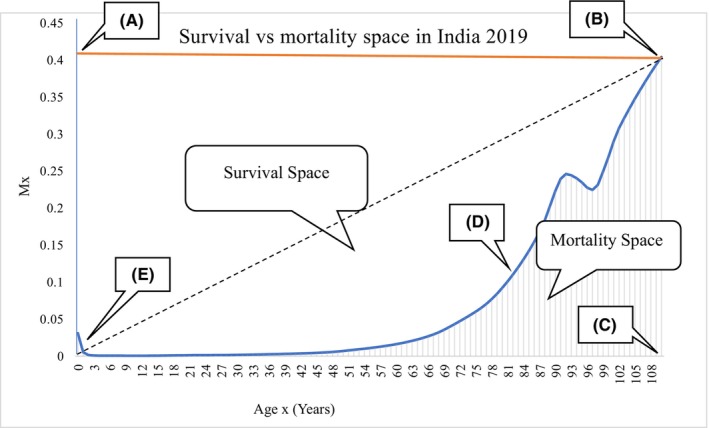
Survival versus mortality space plot of the population in India for 2019 (ABCDE).

Figure 1 shows the survival versus mortality space plot of the population in India for the year 2019. Here, we use the life table data for the calculation of HLYL. The blue exponential curve shows the age specific mortality ($m_{x}$) at age *x*. The Survival‐Mortality Space (SMS) diagram consists of both survival and mortality probability spaces. A similar study conducted by Roman et al.,^21^ where they used the expression of the survival function as *H*(*x*) denotes the cumulative hazard function, which is equivalent to the area under the hazard function $m_{x}$. The area under the hazard function was defined by taking the corresponding integration limits ranging from *x* (current age of an individual) to *x* + $y_{x}$ (age at death or quantity of time lived from birth to death). The calculated area will give the risk of dying at a given age *x* up to a particular future time $y_{x}$. This approach was developed by Skiadas and Skiadas^1^, ^16^, ^19^, ^20^, ^22^, ^23^ to set a time‐varying fraction $b_{x}$ of the form given below:

(1) $$b_{x}=\frac{\text{Total space}\;}{\text{Mortality space}}=\frac{\text{EABCE}\;}{\text{EDBCE}}=\frac{xm_{x}}{S\left(m_{x}\right)}=\frac{xm_{x}}{\int_{0}^{x}m_{s}\mathrm{ds}}=\frac{xd_{x}}{l_{x}\sum_{0}^{x}\left(\frac{d_{x}}{l_{x}}\right)}$$

Accordingly, the mortality process will have two alternatives expressed by simple Equation^16^;

(2) $$xm_{x}=b_{x}\int_{0}^{x}m_{s}\mathrm{ds}\approx b_{x}\sum_{o}^{x}m_{x}$$

#### 2.2.2 Log‐logistic model

The log‐logistic distribution (LLD) is obtained by applying the logarithmic transformation to the logistic distribution (LD). LLD is mathematically more tractable when compared with the log‐normal distribution. For this property, LLD is being used in survival data analysis.^24^ The LLD can also be a good replacement for the Weibull distribution. As it has a closed form of distribution function and its hazard function is quite flexible, this distribution has greater scope and may be applicable to a wide variety of problems in various areas.

Let *X* be a non‐negative random variable that follows log‐logistic distribution with two parameters λ and l. Then, the probability density function *f*(*x*), survival function *S*(*x*), and hazard function *h*(*x*) becomes^25^:

(3) $$f\left(x\right)=\frac{\left(\mathrm{\lambda l}\;x^{l-1}\right)}{{\left(1+{\left(\mathrm{\lambda x}\right)}^{l}\right)}^{2}}$$

(4) $$S\left(x\right)=\frac{1}{\left(1+\lambda x^{l}\right)}$$

(5) $$h\left(x\right)=\frac{\left(\mathrm{\lambda l}\;x^{l-1}\right)}{\left(1+{\left(\mathrm{\lambda x}\right)}^{l}\right)}$$

To derive log‐logistic generating function, here, we take the shape parameter as unity, that is, λ=1, hence we have:

(6) $$b_{x}=\frac{xm_{x}}{\int_{0}^{x}m_{s}\mathrm{ds}}\Rightarrow b_{x}=\frac{x\frac{lx^{l-1}}{\left(1+x^{l}\right)}}{\int_{0}^{x}\frac{ls^{l-1}}{\left(1+s^{l}\right)}\mathrm{ds}}$$

Let $1+s^{l}=u$ $\Rightarrow \mathrm{du}=ls^{l-1}\mathrm{ds}$. Now considering the denominator of the above equation we have:

(7) $$\int_{0}^{x}\frac{ls^{l-1}}{\left(1+s^{l}\right)}\mathrm{ds}=\int_{0}^{x}\frac{1}{u}\mathrm{du}=\left[\ln u\right]\begin{array}{c} x\\ 0 \end{array}=\left[\ln\left(1+s^{l}\right)\right]\begin{array}{c} x\\ 0 \end{array}=\ln\left(1+x^{l}\right)$$

Now,

(8) $$b_{x}=\frac{x\frac{lx^{l-1}}{\left(1+x^{l}\right)}}{\ln\left(1+x^{l}\right)}=\frac{lx^{l}}{\left(1+x^{l}\right)\ln\left(1+x^{l}\right)}$$

Further rewriting Equation (1) we get:

(9) $$\frac{xm_{x}}{b_{x}}=\int_{0}^{x}\mu_{s}\mathrm{ds}$$

Now, putting the value of $b_{x}$ in the above equation we have:

(10) $$\begin{array}{c} \frac{xm_{x}\left(1+x^{l}\right)\ln\left(1+x^{l}\right)}{lx^{l}}=\int_{0}^{x}m_{s}\mathrm{ds}\\ \Rightarrow \frac{\ln\left(1+x^{l}\right)}{m_{x}}=\frac{x\left(1+x^{l}\right)\ln\left(1+x^{l}\right)}{lx^{l}}\\ \begin{array}{c} \Rightarrow m_{x}=\frac{lx^{l}}{x\left(1+x^{l}\right)}\\ \Rightarrow m_{x}=\frac{lx^{l-1}}{\left(1+x^{l}\right)} \end{array} \end{array}$$

Here, $m_{x}$ is the hazard function or generating function of LLD. The selected value for the estimation of the HLYL is provided by the parameter l.

#### 2.2.3 Weibull model

Matsushita et al.,^15^ had suggested the Weibull model for a life time data analysis of disease and aging. It was also established that the Weibull model can be used to estimate the HLYL due to disabilities. Skiadas and Skiadas^16^ highlight the importance of introducing the Weibull shape parameter in connection to survival rates. From the above Equation 1, ^16^ taking $b_{x}$ as constant and the found that the estimator $b_{x}$ follows the hazard function of the Weibull distribution.

Let *X* be a non‐negative random variable that follows the Weibull distribution with two parameters *b* and l. Then, the probability density function *f*(*x*), survival function *S*(*x*), and hazard function *h*(*x*) becomes^25^:

(11) $$f\left(x\right)=\mathrm{bl}{\left(b\;x\right)}^{l-1}\;\exp\left\{-{\left(b\;x\right)}^{l}\right\}$$

(12) $$S\left(x\right)=\exp\left\{-{\left(b\;x\right)}^{l}\right\}$$

(13) $$h\left(x\right)=\mathrm{bl}{\left(b\;x\right)}^{l-1}$$

#### 2.2.4 Gompertz model

In the Gompertz model, Skiadas put $b_{x}$ as a multiple of a constant with age *x*, that is $b_{x}=\mathrm{ax}$ in Equation 1, which is inter‐related with hazard function of the Gompertz distribution.^26^ The Gompertz model is generally used to handle the mortality data. A convenient Gompertz model is provided by Carriere^27^ as $\mu_{x}=Bc^{x}$, where *B* and *c* are the parameters.

This model provides a probability density function *f*(*x*) expressing the distribution of deaths over age for a population at a specific period of time. Then the probability density function *f*(*x*), cumulative distribution function *F*(*x*), survival function *S*(*x*), and hazard function *h*(*x*) becomes:

(14) $$f\left(x\right)=be^{-l+\mathrm{bx}}e^{-e^{-l+\mathrm{bx}}}$$

(15) $$F\left(x\right)=e^{-e^{-l+\mathrm{bx}}}$$

(16) $$S\left(x\right)=1-e^{-e^{-l+\mathrm{bx}}}$$

(17) $$h\left(x\right)=\frac{f\left(x\right)}{F\left(x\right)}=be^{-l+\mathrm{bx}}=e^{\ln\left(b\right)-l+\mathrm{bx}}$$

All the statistical analysis and plotting has been done using two statistical software programs viz., R version 3.6.2 and Microsoft Excel.

## 3 ANALYSIS AND RESULTS

Primarily, HLYL was estimated by using the above discussed four methods viz. Direct, Gompertz, Weibull, and log‐logistic for the total, male, and female populations, respectively, in India from 2000 to 2019 and presented in the Table 2. Note that the direct method given by Skiadas^17^ was based on only life tables and can be used across time periods as long as the life table exists. The results are presented in an illustrative graph and a table in Appendix S1 in part with the growing trend for$\;b_{x}$ to reach a maximum and a decline at higher ages.

**FIGURE 2:**
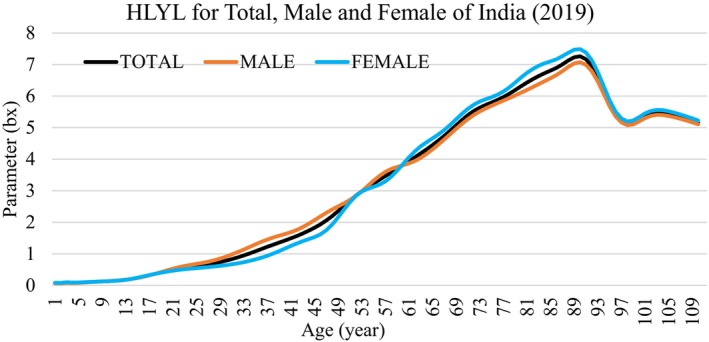
Age specific healthy life years lost (HLYL) among the total, Male, and female populations in India in 2019.

The age specific HLYL values ($b_{x}$) of the total, male, and female populations of India for the year 2019 are plotted in Figure 2. The figure shows, up to 21 years of age for both male and female subjects are having almost the same years of HLYL. But the population with 22 and above years of age are having more or less different HLYL. From the age 22–59 years, HLYL for the male population is quite higher than the female population. But for the age 60 and over, women are dominant over men in India. By looking at overall characteristics of HLYL, as shown in Figure 2, it can be clearly observed that the value of HLYL increases for all three categories of populations, that is, total, male, and female populations up to 92 years of age and decreases on reaching 93 years and above.

HLYL was estimated by using three probabilistic models viz. Gompertz, Weibull, and log‐logistic for the total, male, and female populations, respectively, in India from 2000 to 2019 and the model fit summary of these considered models is given in Table 1.

**TABLE 1:** Model fit summary.

Model	Total			Male			Female		
	$R^{2}$	SSE	Std. Error	$R^{2}$	SSE	Std. Error	$R^{2}$	SSE	Std. Error
Log‐logistic	0.947	0.001	0.003	0.936	0.001	0.003	0.960	0.001	0.003
Weibull	0.900	0.001	0.003	0.892	0.001	0.003	0.909	0.001	0.003
Gompertz	0.918	0.001	0.003	0.913	0.001	0.003	0.922	0.001	0.003

In this study, we have considered three life time models, that is, the log‐logistic, the Weibull, and the Gompertz models to fit age‐specific mortality data for the total Indian population and the male and female populations, respectively. The model summary is given in Table 1, which shows all the three considered models and it gives very similar values of *R* ^2^, SSE, and standard error. For the log‐logistic model, the *R* ^2^ value is found to be maximum for the total, male, and female populations as 0.947, 0.936, and 0.960, respectively. Therefore, the log‐logistic model is considered to be the best fitted model with the greater accuracy.

Table 2 shows an increasing trend of HLYL for the total, male, and female populations in India in all the four discussed methods from 2000 to 2019. The related figures for 2000 are $b_{x}$ = 6.82 with the direct estimate, $b_{x}$ = 5.74 with the Gompertz estimate, $b_{x}$ = 5.88 for the Weibull model, and $b_{x}$ = 7.30 via the log‐logistic model. The related figures for 2019 are $b_{x}$ = 7.26 for direct life table estimation, $b_{x}$ = 6.64 for the Gompertz model, $b_{x}$ = 6.66 for the Weibull model, and $b_{x}$ = 8.79 for the log‐logistic model. Note that the figure for the HLYL provided by the WHO is 10.49 years of age. By comparing the estimated HLYL with the WHO estimates it is found that the log‐logistic model provides more close estimates than the other considered methods. Further, the outcomes of the log‐logistic model shows that the HLYL for women are comparatively higher than their counterparts, that is, the male population in India during 2000 to 2019. From the log‐logistic model, current estimates of HLYL for the male and female populations in India in 2019 becomes 8.36 and 9.35 years, respectively.

**TABLE 2:** HLYL estimates and comparison for the total, male, and female populations in India from 2000 to 2019

Year	Direct			Log‐logistic			Gompertz			Weibull			WHO		
	T	M	F	T	M	F	T	M	F	T	M	F	T	M	F
2000	6.824	6.879	6.900	7.302	7.074	7.641	5.743	5.482	6.090	5.876	5.606	6.230	9.210	8.140	10.220
2001	6.842	6.893	6.916	7.387	7.142	7.742	5.796	5.528	6.202	5.922	5.645	6.279			
2002	6.860	6.906	6.932	7.472	7.211	7.843	5.848	5.573	6.202	5.968	5.684	6.327			
2003	6.879	6.921	6.948	7.559	7.282	7.943	5.901	5.620	6.257	6.014	5.724	6.375			
2004	6.898	6.938	6.964	7.646	7.355	8.043	5.954	5.669	6.311	6.060	5.767	6.422			
2005	6.917	6.956	6.979	7.735	7.431	8.142	6.008	5.720	6.363	6.107	5.812	6.467			
2006	6.937	6.972	6.996	7.823	7.507	8.241	6.061	5.771	6.416	6.154	5.856	6.513			
2007	6.957	6.984	7.016	7.911	7.582	8.341	6.113	5.820	6.470	6.199	5.899	6.560			
2008	6.976	6.987	7.044	7.998	7.653	8.446	6.164	5.863	6.527	6.244	5.937	6.610			
2009	6.995	6.974	7.085	8.085	7.720	8.557	6.213	5.899	6.591	6.286	5.968	6.665			
2010	7.014	6.951	7.136	8.170	7.784	8.671	6.259	5.931	6.660	6.326	5.994	6.724	9.930	8.690	11.340
2011	7.036	6.932	7.191	8.252	7.847	8.780	6.305	5.962	6.727	6.365	6.021	6.782			
2012	7.062	6.928	7.243	8.330	7.911	8.878	6.350	5.998	6.788	6.404	6.052	6.835			
2013	7.095	6.943	7.288	8.405	7.980	8.962	6.397	6.042	6.840	6.445	6.092	6.880			
2014	7.128	6.966	7.329	8.477	8.051	9.036	6.444	6.090	6.885	6.486	6.136	6.920			
2015	7.160	6.991	7.367	8.546	8.120	9.104	6.488	6.139	6.926	6.526	6.180	6.955	10.210	9.010	11.420
2016	7.191	7.016	7.402	8.611	8.187	9.166	6.531	6.185	6.965	6.564	6.223	6.989			
2017	7.219	7.038	7.435	8.673	8.249	9.226	6.571	6.228	7.002	6.600	6.262	7.022			
2018	7.243	7.057	7.464	8.730	8.305	9.285	6.607	6.264	7.038	6.633	6.296	7.054			
2019	7.262	7.072	7.488	8.786	8.357	9.345	6.641	6.297	7.075	6.664	6.326	7.087	10.490	9.220	11.770

Abbreviations: F, female; HLYL, healthy life years lost; M, male; T, total; WHO, World Health Organization.

Further, HLE also been estimated viz. the direct, the Gompertz, the Weibull, and the Log‐logistic models for the total, male, and female populations in India from 2000 to 2019 and presented in Table 3. LE values for the male, female, and total populations were retrieved from the complete life table given by HMD and presented in Table 3. Then, HLE is being calculated by subtracting HLYL from their corresponding LE.

**TABLE 3:** LE, HLE estimates, and comparison for total, male, and female populations in India from 2000 to 2019

Year	LE			Direct			Log‐Logistic			Gompertz			Weibull			WHO		
	T	M	F	T	M	F	T	M	F	T	M	F	T	M	F	T	M	F
2000	62.499	61.728	63.317	55.675	54.849	56.417	55.197	54.654	55.676	56.756	56.246	57.227	56.623	56.122	57.087	53.518	53.584	53.439
2001	62.898	62.117	63.727	56.056	55.224	56.811	55.511	54.974	55.985	57.102	56.589	57.525	56.976	56.472	57.448			
2002	63.297	62.504	64.138	56.437	55.598	57.206	55.825	55.293	56.295	57.449	56.931	57.936	57.329	56.821	57.810			
2003	63.700	62.894	64.550	56.821	55.973	57.602	56.141	55.613	56.607	57.799	57.274	58.293	57.686	57.170	58.175			
2004	64.101	63.289	64.959	57.203	56.351	57.995	56.455	55.934	56.917	58.147	57.619	58.649	58.041	57.522	58.538			
2005	64.502	63.686	65.367	57.585	56.731	58.388	56.767	56.255	57.226	58.494	57.966	59.004	58.395	57.875	58.900	55.288	55.334	55.220
2006	64.907	64.085	65.781	57.970	57.113	58.785	57.084	56.577	57.541	58.846	58.314	59.365	58.753	58.228	59.268			
2007	65.318	64.481	66.207	58.361	57.497	59.190	57.407	56.899	57.865	59.205	58.661	59.737	59.119	58.582	59.647			
2008	65.749	64.881	66.666	58.773	57.895	59.622	57.751	57.228	58.220	59.585	59.018	60.138	59.505	58.945	60.056			
2009	66.214	65.298	67.182	59.219	58.324	60.097	58.129	57.578	58.625	60.001	59.399	60.590	59.928	59.330	60.517			
2010	66.695	65.721	67.731	59.681	58.770	60.595	58.525	57.937	59.060	60.436	59.791	61.071	60.369	59.727	61.007	57.358	57.157	57.565
2011	67.162	66.131	68.265	60.127	59.200	61.074	58.910	58.284	59.485	60.857	60.170	61.538	60.797	60.110	61.483			
2012	67.584	66.508	68.742	60.522	59.580	61.499	59.254	58.596	59.864	61.234	60.510	61.954	61.180	60.455	61.907			
2013	67.952	66.846	69.144	60.858	59.903	61.856	59.547	58.866	60.182	61.555	60.804	62.305	61.507	60.754	62.264			
2014	68.284	67.160	69.497	61.156	60.194	62.168	59.807	59.110	60.460	61.840	61.070	62.612	61.798	61.024	62.577			
2015	68.593	67.458	69.819	61.433	60.466	62.453	60.047	59.337	60.715	62.105	61.319	62.893	62.067	61.278	62.864	58.895	58.365	59.471
2016	68.879	67.735	70.117	61.688	60.720	62.715	60.268	59.549	60.950	62.348	61.550	63.151	62.315	61.513	63.128			
2017	69.146	67.992	70.396	61.927	60.954	62.961	60.473	59.743	61.170	62.575	61.764	63.393	62.546	61.730	63.374			
2018	69.398	68.229	70.663	62.155	61.172	63.199	60.668	59.924	61.378	62.791	61.964	63.624	62.765	61.933	63.609			
2019	69.638	68.451	70.924	62.376	61.378	63.437	60.852	60.094	61.579	62.997	62.154	63.850	62.974	62.125	63.837	60.300	60.300	60.400

Abbreviations: F, female; HLE, healthy life expectancy; LE, life expectancy; M, male; T, total; WHO, World Health Organization.

Table 3 shows an increasing trend of HLE for the total, male, and female populations in India in all the four methods from 2000 to 2019. By comparing the estimated HLE with the WHO estimates it is found that the log‐logistic model provides closer estimates than the other considered methods. Further, the log‐logistic model shows that the HLE for the female population are comparatively higher than the male population in India during 2000 to 2019. From the log‐logistic model, the current estimates of HLE for the total, male, and female populations in India becomes 60.85, 60.09, and 61.58 years, respectively.

**FIGURE 3:**
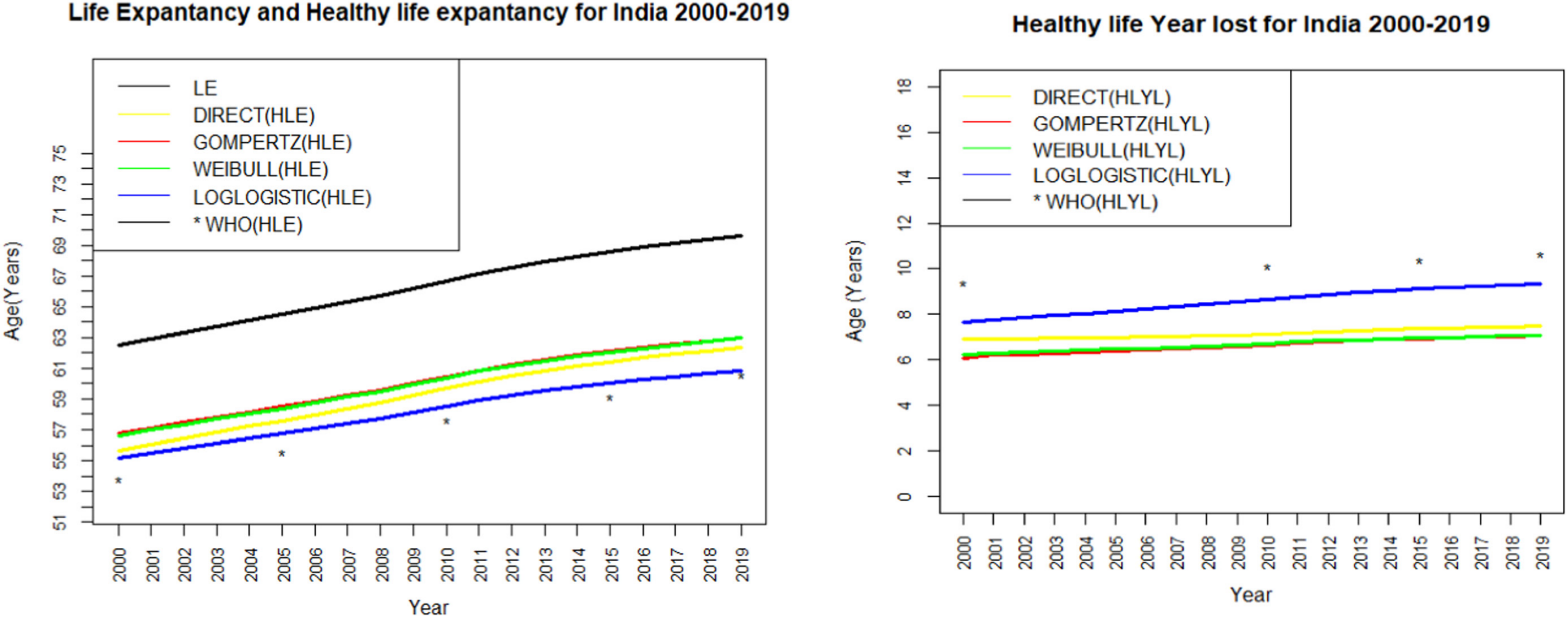
Comparison of life expectancy and healthy life expectancy (left). Comparison of healthy life years lost (right) in India from 2000 to 2019. HLE, healthy life expectancy; HLYL, healthy life years lost; LE, life expectancy; WHO, World Health Organization.

**FIGURE 4:**
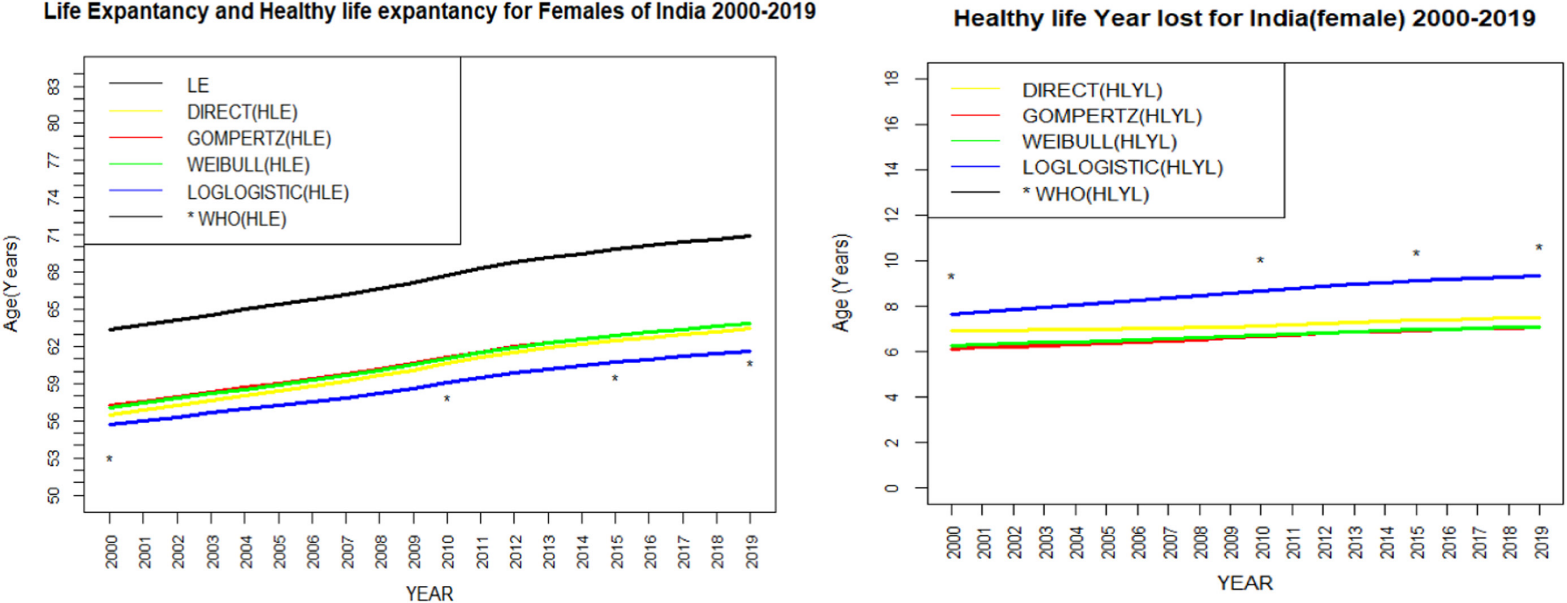
Comparison of life expectancy and healthy life expectancy (left). Comparison of healthy life years lost (right) among women in India from 2000 to 2019. HLE, healthy life expectancy; HLYL, healthy life years lost; LE, life expectancy; WHO, World Health Organization.

**FIGURE 5:**
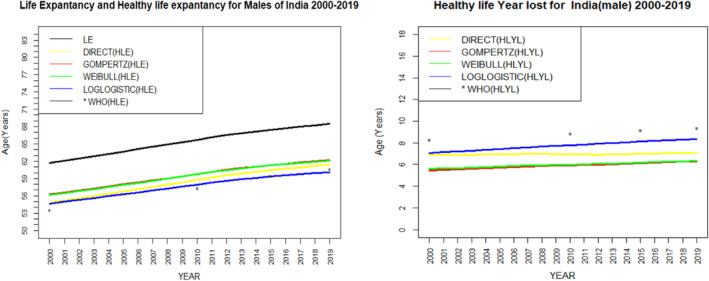
Comparison of life expectancy and healthy life expectancy (left). Comparison of healthy life years lost (right) among men in India from 2000 to 2019. HLE, healthy life expectancy; HLYL, healthy life years lost; LE, life expectancy; WHO, World Health Organization.

Figure 3 shows the graphical comparison between HLE (in the left) and HLYL (in the right) estimated by various methods for India during 2000 to 2019. The figure shows the value of LE and HLE increases over the years. However, the gap between LE and HLE slowly increases as their value increases with the years. As a result, we can see the HLYL also shows an increasing trend over the years. The values of HLE estimated by four different methods viz. the direct, the Weibull, the Gompertz, and the log‐logistic models show nearly similar values. But the estimated value of HLE by the log‐logistic model is closer to the estimated value given by the WHO than the value estimated by the other considered methods or models, which confirms that the log‐logistic model is an appropriate model to estimate HLE for the Indian population. Similarly, for HLYL, the log‐logistic model shows a closer estimate as given by the WHO than the other considered models and confirms as an appropriate model to estimate HLYL for the Indian population. Figures 4 and 5 show the graphical comparison between HLE (in the left) and HLYL (in the right) estimated by various methods for the Indian male and female populations, respectively, during 2000 to 2019. These figures also show an increasing trend of HLE and HLYL for both male and female cases. In addition to this, the log‐logistic model gives more close estimates than the other considered methods or models as compared to the estimates given by the WHO.

## 4 DISCUSSION

In this study, we have used four different techniques viz. the direct, the Weibull, the Gompertz, and the log‐logistic models to estimate HLYL and HLE. The applications of the Weibull and Gompertz models can be found in several studies.^15^, ^19^, ^28^ But the use of the log‐logistic model is not in common with the others. Therefore, in this study, we have proposed and established the analytical explanation of the link between the HLYL ($b_{x}$) parameter and the hazard function of the log‐logistic model. In the same way, Skiadas and Skiadas,^16^ derived a relationship between the Weibull shape parameter and HLYL and applied it on the Japanese population to estimate their HLYL. They have found that the direct method and the Weibull method provides nearly the same estimates as provided by the WHO. Skiadas and Skiadas,^22^ developed a health mortality approach to estimate HLYL of United States and the Japanese populations using the Weibull and Gompertz models and they have also found estimates of HLYL which are very close to the one provided by the WHO.

In our study, we have found an increasing trend of HLE and HLYL over the years irrespective of the gender. Similar findings have been observed by Lau et al.,^29^ an increasing trend of LE and HLE in all age groups as well as for men and women in India over the period 2007 to 2020. In their study, the largest gain in LE and HLE is observed in the age 70+ years among women and the age 65+ years among men. However, in this study, the largest gain in HLE and HLYL observed in the age 90+ years among both men and women. Further, it is also found that there is a significant difference between men and women with respect to their LE and HLE and similar findings were also found in a recent study in India by Borah.^9^ In this study, they have reported that the women have a higher LE than men, which is also observed around the world.^30^

The coronavirus disease 2019 (COVID‐19) pandemic has had a devastating impact on LE and HLYL. In 2020, LE at birth declined by an average of 1.33 years in 27 countries. This means that people born in 2020 are expected to live 1.33 years less than people born in 2019. The pandemic has also had a disproportionate impact on the elderly, with LE at age 65 years declining by an average of 0.9 years in 27 countries.^31^ Brazil's life expectancy at birth declined by 1.3 years in 2020 due to COVID‐19, reaching a level not seen since 2014. The LE at age 65 years also declined by 0.9 years, setting Brazil back to 2012 levels.^32^

Not only the proposed model, that is, the log‐logistic model, provides a good and close estimate of HLYL, the Gompertz and Weibull models also provide good estimates as given by the WHO. Here, in Appendix S1, we have provided the analytical explanation of the relationship between HLYL and the modified Weibull model. This explanation can be used for future study in demography and population health research. Researchers may further extend this model to estimate the HLYL due to COVID‐19 in India.

## 5 CONCLUSION

The evidence presented indicates that the proportion of healthy years in a person's life has remained relatively stable, suggesting that the additional years gained are generally characterized by poor health. If individuals can enjoy these extra years in good health, and if they are supported by an enabling environment, their ability to engage in activities they value may not significantly differ from that of younger individuals. However, if these additional years are primarily marked by physical and mental decline, it has more negative implications for both older individuals and society as a whole.

Furthermore, our findings revealed that HLYL are consistently higher for women compared to men, thus compensating for the longer LE observed in women. As LE increases, there is a corresponding rise in the number of healthy years lost. It is crucial to address this issue by not only extending the lifespan but also by implementing strategies to reduce the HLYL. Moreover, it is essential to enhance the social security system, which often relies solely on LE measures, by incorporating healthy life year data into relevant plans and policies. Interestingly, although women tend to have higher healthy LE compared to their male counterparts, the gap between male and female LE at birth is larger than the gap in healthy LE.

The health care system faces a challenge in adapting its support to the growing segment of the population that surpasses the healthy LE threshold. The concepts of healthy LE and HLYL can serve as valuable indicators for health care resource planning and future policy development in India. Monitoring healthy LE could provide valuable tools for crafting health care strategies aimed at postponing morbidity and disabilities for the entire population. Given that India is a welfare state with a dynamic demographic dividend and a rapidly growing population, addressing concerns related to the aging population in a timely manner is crucial to ensure that the demographic dividend does not transform into a demographic disaster.

Calculating healthy LE at a national level could yield policy recommendations that improve health practices and enhance access to quality health care for the elderly. We hope that this paper will stimulate further research and foster meaningful dialogue on the topic of healthy LE.

The loss of LE and healthy life years is not just a statistical measure. It represents the loss of real lives and real opportunities. It means that people are dying younger and that they are spending more of their lives in poor health. The COVID‐19 pandemic is a reminder of the fragility of life and the importance of taking steps to protect our health.

## AUTHOR CONTRIBUTIONS

*Performed the analysis:* Jena and Tripathy. *Wrote the first draft:* Swain. *Edited and revised the manuscript:* Sarangi. *Developed the idea:* Swain. *Guided the writing of the paper:* Sarangi. All authors read and approved the final manuscript.

## FUNDING INFORMATION

No funding was received for conducting this study.

## CONFLICT OF INTEREST STATEMENT

The authors declare that they have no conflict of interest.

## Supporting information


Appendix S1.

## Data Availability

The datasets used and analyzed in this study are available in the public domain in the website http://www.mortalitytrends.org.

## References

[1] Skiadas CH , Skiadas C . Exploring the Health State of a Population by Dynamic Modeling Methods. The Springer Series on Demographic Methods and Population Analysis 45. Springer; 2018a. doi:10.1007/978-3-319-65142-2

[2] Sullivan DF . A single index of mortality and morbidity. HSMHA Health Rep. 1971;86(4):347‐354. doi:10.2307/4594169 5554262 PMC1937122

[3] World Health Statistics Overview . Monitoring Health for the SDGs, Sustainable Development Goals. World Health Organization; 2019. (WHO/DAD/2019.1). Licence: CC BY‐NC‐SA 3.0 IGO.

[4] Callahan D . The WHO definition of “health”. Stud Hastings Cent. 1973;1(3):77‐88. doi:10.2307/3527467 4607284

[5] National Institute on Aging . Why population aging matters (2007): a global perspective. https://www.nia.nih.gov/sites/default/files/2017‐06/WPAM.pdf

[6] McNicoll G . World population ageing 1950‐2050. Popul Dev Rev. 2002;28(4):814‐816.

[7] Johnson CS , Stevens A , Rajan I . Promotion of healthy aging in the context of population aging phenomenon: a look at the aging state in India. Indian J Gerontol. 2005;19:181‐192.

[8] Riley JC . The timing and pace of health transitions around the world. Popul Dev Rev. 2005;31(4):741‐764. doi:10.1111/j.1728-4457.2005.00096.x

[9] Borah G . Gender gap in life expectancy in India and role of age groups: a comparison between before and after male – female life expectancy at birth crossover. PLoS One. 2021;16(12):e0260657. doi:10.1371/jornal.pone.0260657 34855808 PMC8638908

[10] Sample Registration System (SRS)‐Abridged Life Tables 2015–2019, Office of the Registrar General & Census Commissioner, India (ORGI). SRS‐Abridged_Life_Tables_2015‐2019.pdf.

[11] WHO, Life expectancy and Healthy life expectancy Data by country (2021). https://apps.who.int/gho/data/view.main.SDG2016LEXREGv?lang=en

[12] Jagger C , Van H , Robine JM . Health Expectancy Calculation by the Sullivan Method: A Practical Guide. Institute for Ageing, Newcastle University; 2014. doi:10.1016/S0140-6736(15)00947-2

[13] Romero DE , Leite IC , Szwarcwald CL . Healthy life expectancy in Brazil: applying the Sullivan method. SciELO ‐ Scientific Electronic Library Online. Cad Saude Publica. 2005;21(suppl 1):S7‐S18. doi:10.1590/S0102-311X2005000700002 16462992

[14] Tokudome S , Hashimoto S , Igata A . Life expectancy and healthy life expectancy of Japan: the fastest graying society in the world. BMC Res Notes. 2016;9:482. doi:10.1186/s13104-016-228-2 27793196 PMC5084424

[15] Matsushita S , Hagiwara K , Shiota T , Shimada H , Kuramoto K , Toyokura Y . Lifetime data analysis of disease and aging by the Weibull probability distribution. J Clin Epidemiol. 1992;45(10):1165‐1175. doi:10.1016/0895-4356(92)90157-I 1474413

[16] Skiadas CH , Skiadas C . Direct healthy life expectancy estimates from life tables with a Sullivan extension. Bridging the gap between HALE and Eurostat estimates. In: Lynch S. M. , eds. Demography of Population Health, Aging and Health Expenditures. The Springer Series on Demographic Methods and Population Analysis. Vol 50. Springer; 2020a:25‐42. doi:10.1007/978-3-030-44695-6_3

[17] Skiadas CH , Skiadas C , Dimotikalis Y . Estimating the healthy life expectancy (HLE) in the far past. The case of France (1816‐2017) and comparisons with HALE from WHO and projections to 2060. 2020. doi:10.1007/978-3-030-44695-6

[18] Skiadas CH , Skiadas C . Estimating the healthy life expectancy (HLE) in the far past: the case of Sweden (1751–2016) with forecasts to 2060. In: Dimotikalis Y. , Karagrigoriou A. , Parpoula C. , Skiadas C. H. , eds. Applied Modeling Techniques and Data Analysis 2: Financial, Demographic, Stochastic and Statistical Models and Methods. Vol 8. ISTE Ltd and John Wiley & Sons, Inc; 2021:91‐96. doi:10.1007/978-3-030-44695-6

[19] Skiadas CH , Skiadas C . Relation of the Weibull shape parameter with the healthy life years lost estimates: analytical derivation and estimation from an extended life table. In: Lynch S. M. , eds. The Springer Series on Demographic Methods and Population Analysis 50. Springer; 2020b: 9‐23. doi:10.1007/978-3-030-44695-6_2

[20] Skiadas CH , Skiadas C . How the unsolved problem of finding the Healthy Life Expectancy (HLE) in the far past was resolved: The case of Sweden (1751‐2016) with forecasts to 2060 and comparisons with HALE. 2020c. doi:10.31235/osf.io/akf8v

[21] Román R , Comas M , Hoffmeister L , Castells X . Determining the lifetime density function using a continuous approach. J Epidemiol Community Health. 2007;61(10):923‐925. doi:10.1136/jech.2006.052639 17873232 PMC2652977

[22] Skiadas CH , Skiadas C . The health‐mortality approach in estimating the healthy life years lost compared to the global burden of disease studies and applications in world, USA and Japan. In: Lynch S. M. , eds. Exploring the Health State of a Population by Dynamic Modeling Methods. The Springer Series on Demographic Methods and Population Analysis 45. Springer; 2018b:67‐124.

[23] Skiadas CH , Skiadas C . Demography and Health Issues: Population Aging, Mortality and Data Analysis. The Springer Series on Demographic Methods and Population Analysis 46. Springer; 2018c. doi:10.1007/978-3-319-76002-5

[24] Bennett S . Log‐logistic regression models for survival data. J R Stat Soc Ser C Appl Stat. 1983;32(2):165‐171. doi:10.2307/2347295

[25] Wienke A . Frailty Models in Survival Analysis. Chapman and Hall/CRC; 2010. doi:10.1201/9781420073911

[26] Gompertz B . XXIV. On the nature of the function expressive of the law of human mortality, and on a new mode of determining the value of life contingencies. In a letter to Francis Baily, Esq. FRS &c. Philos Trans R Soc Lond. 1825;115:513‐583. doi:10.1098/rspl.1815.0271 PMC4360127 25750242

[27] Carriere JF . Parametric models for life tables. Transactions of Society of Actuaries. 1992;44:77‐99.

[28] Skiadas CH , Skiadas C . Demography and Health Issues, the Springer Series on Demographic Methods and Population Analysis 46. Springer International Publishing AG; 2018d. doi:10.1007/978-3-319-76002-5_3

[29] Lau RS , Johnson S , Kamalanabhan TJ . Healthy life expectancy in the context of population health and ageing in India. Asia Pac J Public Health. 2012;24(1):195‐207. 20685664 10.1177/1010539510376663

[30] United Nations, Department of Economic and Social Affairs . World Population Ageing 2007. United Nations Publication; 2007. ISBN 978‐92‐1‐151432‐2.

[31] Huang G , Guo F , Zimmermann KF , et al. The effect of the COVID‐19 pandemic on life expectancy in 27 countries. Sci Rep. 2023;13(1):8911. doi:10.1038/s41598-023-35592-9 37264048 PMC10233553

[32] Castro MC , Gurzenda S , Turra CM , Kim S , Andrasfay T , Goldman N . Reduction in life expectancy in Brazil after COVID‐19. Nat Med. 2021;27(9):1629‐1635. doi:10.1038/s41591-021-01437-z 34188224 PMC8446334

