# Supplementary material for: Statistical modeling and estimating number of healthy life years lost and healthy life expectancy in India, 2000–2019
Source: Aging Med (Milton). 2023 Sep 27;6(4):435–45. doi: 10.1002/agm2.12269 (PMC10792328; doi:10.1002/agm2.12269)
Supplement: Supplementary file 1 — Appendix S1. [file AGM2-6-435-s001.docx]

**Appendix S1**

Table A1: Estimation of HLYL by using Direct method (2019)

| Age | mx | qx | ax | lx | dx | Lx | ex | Μx | M ̅x | PLYL | bx | HLE |
| --- | --- | --- | --- | --- | --- | --- | --- | --- | --- | --- | --- | --- |
| 0 | 0.0310 | 0.0302 | 0.13 | 100000 | 3022.83 | 97377.29 | 69.638 | 0.030 | 0.130 | 23236.6 | 0.23 | 69.41 |
| 1 | 0.0052 | 0.0052 | 0.5 | 96977.17 | 503.08 | 96725.63 | 70.805 | 0.035 | 0.024 | 21307 | 0.18 | 70.62 |
| 2 | 0.0014 | 0.0014 | 0.5 | 96474.08 | 139.62 | 96404.27 | 70.171 | 0.037 | 0.015 | 9469 | 0.14 | 70.03 |
| 3 | 0.0009 | 0.0009 | 0.5 | 96334.46 | 84.75 | 96292.09 | 69.272 | 0.038 | 0.011 | 7859 | 0.11 | 69.16 |
| 4 | 0.0007 | 0.0007 | 0.5 | 96249.71 | 68.22 | 96215.6 | 68.333 | 0.038 | 0.009 | 7984 | 0.09 | 68.24 |
| 5 | 0.0007 | 0.0007 | 0.5 | 96181.49 | 62.9 | 96150.04 | 67.381 | 0.039 | 0.007 | 8847 | 0.10 | 67.28 |
| 6 | 0.0007 | 0.0007 | 0.5 | 96118.59 | 62.88 | 96087.15 | 66.425 | 0.040 | 0.006 | 10280 | 0.11 | 66.32 |
| 7 | 0.0006 | 0.0006 | 0.5 | 96055.71 | 62.12 | 96024.65 | 65.468 | 0.040 | 0.005 | 11530 | 0.12 | 65.35 |
| 8 | 0.0006 | 0.0006 | 0.5 | 95993.59 | 58.88 | 95964.15 | 64.510 | 0.041 | 0.005 | 12201 | 0.13 | 64.38 |
| 9 | 0.0006 | 0.0006 | 0.5 | 95934.71 | 56.26 | 95906.58 | 63.549 | 0.042 | 0.004 | 12846 | 0.13 | 63.42 |
| 10 | 0.0006 | 0.0006 | 0.5 | 95878.45 | 54.32 | 95851.29 | 62.586 | 0.042 | 0.004 | 13524 | 0.14 | 62.44 |
| 11 | 0.0006 | 0.0006 | 0.5 | 95824.13 | 53.12 | 95797.57 | 61.622 | 0.043 | 0.004 | 14297 | 0.15 | 61.47 |
| 12 | 0.0006 | 0.0006 | 0.5 | 95771.01 | 52.76 | 95744.63 | 60.655 | 0.043 | 0.003 | 15239 | 0.17 | 60.49 |
| 13 | 0.0006 | 0.0006 | 0.5 | 95718.25 | 54.84 | 95690.83 | 59.689 | 0.044 | 0.003 | 16883 | 0.19 | 59.50 |
| 14 | 0.0006 | 0.0006 | 0.5 | 95663.41 | 60.34 | 95633.24 | 58.723 | 0.044 | 0.003 | 19669 | 0.22 | 58.51 |
| 15 | 0.0007 | 0.0007 | 0.5 | 95603.07 | 68.42 | 95568.86 | 57.759 | 0.045 | 0.003 | 23464 | 0.25 | 57.51 |
| 16 | 0.0008 | 0.0008 | 0.5 | 95534.66 | 77.86 | 95495.72 | 56.800 | 0.046 | 0.003 | 27920 | 0.29 | 56.51 |
| 17 | 0.0009 | 0.0009 | 0.5 | 95456.79 | 86.55 | 95413.52 | 55.846 | 0.047 | 0.003 | 32282 | 0.34 | 55.51 |
| 18 | 0.0010 | 0.0010 | 0.5 | 95370.24 | 94.25 | 95323.12 | 54.896 | 0.048 | 0.003 | 36396 | 0.38 | 54.51 |
| 19 | 0.0011 | 0.0011 | 0.5 | 95275.99 | 102.84 | 95224.57 | 53.950 | 0.049 | 0.003 | 40937 | 0.43 | 53.52 |
| 20 | 0.0012 | 0.0012 | 0.5 | 95173.15 | 111.6 | 95117.35 | 53.008 | 0.050 | 0.002 | 45611 | 0.48 | 52.53 |
| 21 | 0.0013 | 0.0013 | 0.5 | 95061.55 | 119.59 | 95001.76 | 52.070 | 0.051 | 0.002 | 50006 | 0.52 | 51.55 |
| 22 | 0.0013 | 0.0013 | 0.5 | 94941.97 | 125.6 | 94879.17 | 51.135 | 0.053 | 0.002 | 53583 | 0.56 | 50.58 |
| 23 | 0.0014 | 0.0014 | 0.5 | 94816.37 | 129.52 | 94751.61 | 50.202 | 0.054 | 0.002 | 56254 | 0.59 | 49.61 |
| 24 | 0.0014 | 0.0014 | 0.5 | 94686.85 | 132.44 | 94620.63 | 49.270 | 0.056 | 0.002 | 58459 | 0.61 | 48.66 |
| 25 | 0.0014 | 0.0014 | 0.5 | 94554.41 | 134.47 | 94487.18 | 48.338 | 0.057 | 0.002 | 60235 | 0.64 | 47.70 |
| 26 | 0.0014 | 0.0014 | 0.5 | 94419.94 | 136.43 | 94351.73 | 47.406 | 0.058 | 0.002 | 61937 | 0.66 | 46.74 |
| 27 | 0.0015 | 0.0015 | 0.5 | 94283.52 | 139.83 | 94213.6 | 46.474 | 0.060 | 0.002 | 64244 | 0.69 | 45.78 |
| 28 | 0.0015 | 0.0015 | 0.5 | 94143.69 | 145.1 | 94071.14 | 45.542 | 0.061 | 0.002 | 67355 | 0.72 | 44.82 |
| 29 | 0.0016 | 0.0016 | 0.5 | 93998.59 | 151.82 | 93922.68 | 44.612 | 0.063 | 0.002 | 71077 | 0.76 | 43.85 |
| 30 | 0.0017 | 0.0017 | 0.5 | 93846.76 | 159.81 | 93766.86 | 43.683 | 0.065 | 0.002 | 75319 | 0.81 | 42.88 |
| 31 | 0.0018 | 0.0018 | 0.5 | 93686.96 | 168.82 | 93602.55 | 42.757 | 0.067 | 0.002 | 79948 | 0.86 | 41.90 |
| 32 | 0.0019 | 0.0019 | 0.5 | 93518.14 | 178.57 | 93428.86 | 41.833 | 0.068 | 0.002 | 84815 | 0.91 | 40.92 |
| 33 | 0.0020 | 0.0020 | 0.5 | 93339.57 | 189.67 | 93244.74 | 40.912 | 0.070 | 0.002 | 90181 | 0.97 | 39.94 |
| 34 | 0.0022 | 0.0022 | 0.5 | 93149.91 | 202.71 | 93048.55 | 39.994 | 0.073 | 0.002 | 96284 | 1.04 | 38.96 |
| 35 | 0.0023 | 0.0023 | 0.5 | 92947.2 | 217.23 | 92838.58 | 39.081 | 0.075 | 0.002 | 102862 | 1.11 | 37.97 |
| 36 | 0.0025 | 0.0025 | 0.5 | 92729.97 | 232.61 | 92613.66 | 38.171 | 0.077 | 0.002 | 109580 | 1.18 | 36.99 |
| 37 | 0.0027 | 0.0027 | 0.5 | 92497.36 | 248.53 | 92373.09 | 37.266 | 0.080 | 0.002 | 116256 | 1.25 | 36.02 |
| 38 | 0.0029 | 0.0028 | 0.5 | 92248.83 | 262.81 | 92117.42 | 36.365 | 0.083 | 0.002 | 121883 | 1.32 | 35.05 |
| 39 | 0.0030 | 0.0030 | 0.5 | 91986.01 | 277.54 | 91847.24 | 35.467 | 0.086 | 0.002 | 127426 | 1.39 | 34.08 |
| 40 | 0.0032 | 0.0032 | 0.5 | 91708.47 | 292.77 | 91562.09 | 34.573 | 0.089 | 0.002 | 132891 | 1.45 | 33.12 |
| 41 | 0.0034 | 0.0034 | 0.5 | 91415.71 | 309.26 | 91261.07 | 33.682 | 0.093 | 0.002 | 138587 | 1.52 | 32.16 |
| 42 | 0.0036 | 0.0036 | 0.5 | 91106.44 | 327.94 | 90942.47 | 32.795 | 0.096 | 0.002 | 144868 | 1.60 | 31.20 |
| 43 | 0.0039 | 0.0038 | 0.5 | 90778.51 | 348.93 | 90604.04 | 31.911 | 0.100 | 0.002 | 151706 | 1.68 | 30.23 |
| 44 | 0.0041 | 0.0041 | 0.5 | 90429.58 | 372.01 | 90243.57 | 31.033 | 0.104 | 0.002 | 158925 | 1.76 | 29.27 |
| 45 | 0.0044 | 0.0044 | 0.5 | 90057.56 | 397.8 | 89858.66 | 30.159 | 0.109 | 0.002 | 166693 | 1.86 | 28.30 |
| 46 | 0.0048 | 0.0048 | 0.5 | 89659.76 | 427.03 | 89446.25 | 29.290 | 0.113 | 0.002 | 175190 | 1.97 | 27.32 |
| 47 | 0.0052 | 0.0052 | 0.5 | 89232.74 | 460.6 | 89002.44 | 28.428 | 0.119 | 0.002 | 184618 | 2.09 | 26.34 |
| 48 | 0.0057 | 0.0057 | 0.5 | 88772.14 | 501.77 | 88521.26 | 27.573 | 0.124 | 0.003 | 196005 | 2.23 | 25.34 |
| 49 | 0.0063 | 0.0063 | 0.5 | 88270.37 | 552.21 | 87994.27 | 26.727 | 0.130 | 0.003 | 209595 | 2.39 | 24.34 |
| 50 | 0.0070 | 0.0070 | 0.5 | 87718.16 | 610.05 | 87413.14 | 25.892 | 0.137 | 0.003 | 224267 | 2.56 | 23.34 |
| 51 | 0.0077 | 0.0077 | 0.5 | 87108.11 | 672.09 | 86772.07 | 25.070 | 0.145 | 0.003 | 238567 | 2.72 | 22.35 |
| 52 | 0.0085 | 0.0085 | 0.5 | 86436.02 | 733.33 | 86069.35 | 24.261 | 0.154 | 0.003 | 250700 | 2.88 | 21.38 |
| 53 | 0.0093 | 0.0092 | 0.5 | 85702.69 | 791.64 | 85306.87 | 23.464 | 0.163 | 0.003 | 260141 | 3.02 | 20.44 |
| 54 | 0.0101 | 0.0100 | 0.5 | 84911.05 | 849.83 | 84486.13 | 22.678 | 0.173 | 0.003 | 268007 | 3.15 | 19.53 |
| 55 | 0.0109 | 0.0108 | 0.5 | 84061.22 | 909.21 | 83606.61 | 21.903 | 0.184 | 0.003 | 274796 | 3.27 | 18.64 |
| 56 | 0.0118 | 0.0117 | 0.5 | 83152 | 971.49 | 82666.26 | 21.137 | 0.195 | 0.003 | 281030 | 3.38 | 17.75 |
| 57 | 0.0127 | 0.0126 | 0.5 | 82180.51 | 1038.81 | 81661.11 | 20.381 | 0.208 | 0.004 | 287233 | 3.50 | 16.88 |
| 58 | 0.0138 | 0.0137 | 0.5 | 81141.7 | 1110.74 | 80586.33 | 19.635 | 0.222 | 0.004 | 293165 | 3.61 | 16.02 |
| 59 | 0.0149 | 0.0148 | 0.5 | 80030.96 | 1185.69 | 79438.11 | 18.901 | 0.236 | 0.004 | 298354 | 3.73 | 15.17 |
| 60 | 0.0162 | 0.0160 | 0.5 | 78845.27 | 1264.46 | 78213.04 | 18.177 | 0.252 | 0.004 | 302974 | 3.84 | 14.33 |
| 61 | 0.0175 | 0.0174 | 0.5 | 77580.81 | 1347.9 | 76906.86 | 17.465 | 0.270 | 0.004 | 307169 | 3.96 | 13.50 |
| 62 | 0.0190 | 0.0189 | 0.5 | 76232.91 | 1437.01 | 75514.4 | 16.765 | 0.289 | 0.005 | 311072 | 4.08 | 12.68 |
| 63 | 0.0207 | 0.0205 | 0.5 | 74795.9 | 1531.37 | 74030.21 | 16.078 | 0.309 | 0.005 | 314501 | 4.21 | 11.87 |
| 64 | 0.0225 | 0.0223 | 0.5 | 73264.53 | 1630.2 | 72449.43 | 15.404 | 0.331 | 0.005 | 317240 | 4.33 | 11.07 |
| 65 | 0.0245 | 0.0242 | 0.5 | 71634.33 | 1734.04 | 70767.31 | 14.743 | 0.356 | 0.005 | 319355 | 4.46 | 10.28 |
| 66 | 0.0267 | 0.0264 | 0.5 | 69900.29 | 1843.5 | 68978.54 | 14.096 | 0.382 | 0.006 | 320902 | 4.60 | 9.50 |
| 67 | 0.0292 | 0.0288 | 0.5 | 68056.78 | 1959.23 | 67077.17 | 13.464 | 0.411 | 0.006 | 321917 | 4.75 | 8.72 |
| 68 | 0.0321 | 0.0316 | 0.5 | 66097.55 | 2087.58 | 65053.76 | 12.849 | 0.442 | 0.006 | 323237 | 4.91 | 7.94 |
| 69 | 0.0355 | 0.0348 | 0.5 | 64009.97 | 2229.62 | 62895.16 | 12.251 | 0.477 | 0.007 | 324704 | 5.07 | 7.18 |
| 70 | 0.0392 | 0.0385 | 0.5 | 61780.35 | 2377.64 | 60591.53 | 11.675 | 0.516 | 0.007 | 325031 | 5.24 | 6.44 |
| 71 | 0.0432 | 0.0423 | 0.5 | 59402.71 | 2509.74 | 58147.84 | 11.123 | 0.558 | 0.008 | 321609 | 5.38 | 5.74 |
| 72 | 0.0472 | 0.0462 | 0.5 | 56892.98 | 2625.61 | 55580.17 | 10.591 | 0.604 | 0.008 | 315100 | 5.51 | 5.08 |
| 73 | 0.0514 | 0.0501 | 0.5 | 54267.37 | 2718.07 | 52908.33 | 10.080 | 0.654 | 0.009 | 305377 | 5.60 | 4.48 |
| 74 | 0.0556 | 0.0541 | 0.5 | 51549.3 | 2790.06 | 50154.27 | 9.585 | 0.708 | 0.010 | 293452 | 5.68 | 3.90 |
| 75 | 0.0601 | 0.0584 | 0.5 | 48759.24 | 2846.48 | 47336 | 9.105 | 0.767 | 0.010 | 280303 | 5.75 | 3.35 |
| 76 | 0.0651 | 0.0630 | 0.5 | 45912.75 | 2892.71 | 44466.4 | 8.638 | 0.830 | 0.011 | 266711 | 5.82 | 2.81 |
| 77 | 0.0706 | 0.0682 | 0.5 | 43020.05 | 2934.1 | 41553 | 8.185 | 0.898 | 0.012 | 253246 | 5.91 | 2.28 |
| 78 | 0.0770 | 0.0741 | 0.5 | 40085.95 | 2972.08 | 38599.91 | 7.748 | 0.972 | 0.012 | 240016 | 6.00 | 1.75 |
| 79 | 0.0842 | 0.0808 | 0.5 | 37113.87 | 2999.26 | 35614.24 | 7.328 | 1.053 | 0.013 | 226469 | 6.11 | 1.22 |
| 80 | 0.0922 | 0.0882 | 0.5 | 34114.61 | 3007.52 | 32610.85 | 6.928 | 1.141 | 0.014 | 212182 | 6.21 | 0.72 |
| 81 | 0.1009 | 0.0961 | 0.5 | 31107.09 | 2989.1 | 29612.54 | 6.550 | 1.237 | 0.015 | 196919 | 6.32 | 0.23 |
| 82 | 0.1102 | 0.1045 | 0.5 | 28118 | 2937.27 | 26649.36 | 6.193 | 1.342 | 0.016 | 180627 | 6.41 | -0.22 |
| 83 | 0.1201 | 0.1133 | 0.5 | 25180.73 | 2852.37 | 23754.54 | 5.857 | 1.455 | 0.017 | 163709 | 6.49 | -0.63 |
| 84 | 0.1307 | 0.1227 | 0.5 | 22328.35 | 2738.92 | 20958.9 | 5.541 | 1.578 | 0.019 | 146710 | 6.56 | -1.02 |
| 85 | 0.1421 | 0.1326 | 0.5 | 19589.44 | 2598.29 | 18290.29 | 5.246 | 1.710 | 0.020 | 129903 | 6.62 | -1.38 |
| 86 | 0.1542 | 0.1432 | 0.5 | 16991.15 | 2433.01 | 15774.64 | 4.972 | 1.853 | 0.021 | 113554 | 6.69 | -1.72 |
| 87 | 0.1672 | 0.1543 | 0.5 | 14558.14 | 2246.75 | 13434.77 | 4.719 | 2.008 | 0.023 | 97919 | 6.77 | -2.05 |
| 88 | 0.1833 | 0.1679 | 0.5 | 12311.39 | 2066.79 | 11278 | 4.489 | 2.176 | 0.025 | 84075 | 6.86 | -2.38 |
| 89 | 0.2030 | 0.1843 | 0.5 | 10244.6 | 1887.85 | 9300.68 | 4.294 | 2.360 | 0.026 | 71599 | 6.94 | -2.64 |
| 90 | 0.2232 | 0.2008 | 0.5 | 8356.75 | 1678.15 | 7517.68 | 4.151 | 2.561 | 0.028 | 59310 | 6.95 | -2.80 |
| 91 | 0.2394 | 0.2138 | 0.5 | 6678.61 | 1428.16 | 5964.53 | 4.069 | 2.774 | 0.030 | 47099 | 6.85 | -2.78 |
| 92 | 0.2461 | 0.2191 | 0.5 | 5250.45 | 1150.47 | 4675.21 | 4.040 | 2.994 | 0.032 | 35549 | 6.64 | -2.60 |
| 93 | 0.2443 | 0.2177 | 0.5 | 4099.97 | 892.75 | 3653.6 | 4.033 | 3.211 | 0.034 | 25993 | 6.32 | -2.29 |
| 94 | 0.2402 | 0.2145 | 0.5 | 3207.22 | 687.89 | 2863.28 | 4.016 | 3.426 | 0.036 | 18975 | 5.94 | -1.92 |
| 95 | 0.2347 | 0.2101 | 0.5 | 2519.33 | 529.21 | 2254.73 | 3.977 | 3.636 | 0.038 | 13900 | 5.55 | -1.58 |
| 96 | 0.2274 | 0.2042 | 0.5 | 1990.12 | 406.43 | 1786.91 | 3.901 | 3.840 | 0.040 | 10213 | 5.25 | -1.35 |
| 97 | 0.2245 | 0.2018 | 0.5 | 1583.69 | 319.64 | 1423.87 | 3.774 | 4.042 | 0.041 | 7710 | 5.05 | -1.28 |
| 98 | 0.2312 | 0.2073 | 0.5 | 1264.05 | 262.01 | 1133.05 | 3.602 | 4.249 | 0.043 | 6074 | 4.96 | -1.36 |
| 99 | 0.2494 | 0.2218 | 0.5 | 1002.04 | 222.21 | 890.94 | 3.413 | 4.471 | 0.045 | 4945 | 4.97 | -1.56 |
| 100 | 0.2690 | 0.2371 | 0.5 | 779.83 | 184.9 | 687.38 | 3.243 | 4.708 | 0.047 | 3947 | 5.04 | -1.80 |
| 101 | 0.2903 | 0.2535 | 0.5 | 594.93 | 150.8 | 519.53 | 3.095 | 4.962 | 0.049 | 3085 | 5.13 | -2.03 |
| 102 | 0.3081 | 0.2670 | 0.5 | 444.12 | 118.57 | 384.84 | 2.976 | 5.229 | 0.051 | 2324 | 5.17 | -2.20 |
| 103 | 0.3219 | 0.2773 | 0.5 | 325.56 | 90.26 | 280.42 | 2.878 | 5.506 | 0.053 | 1697 | 5.19 | -2.31 |
| 104 | 0.3351 | 0.2870 | 0.5 | 235.29 | 67.53 | 201.53 | 2.790 | 5.793 | 0.055 | 1218 | 5.17 | -2.38 |
| 105 | 0.3477 | 0.2962 | 0.5 | 167.76 | 49.7 | 142.92 | 2.712 | 6.089 | 0.058 | 861 | 5.12 | -2.41 |
| 106 | 0.3598 | 0.3050 | 0.5 | 118.07 | 36.01 | 100.06 | 2.643 | 6.394 | 0.060 | 600 | 5.07 | -2.43 |
| 107 | 0.3715 | 0.3133 | 0.5 | 82.06 | 25.71 | 69.21 | 2.583 | 6.707 | 0.062 | 412 | 5.02 | -2.43 |
| 108 | 0.3827 | 0.3212 | 0.5 | 56.35 | 18.1 | 47.3 | 2.534 | 7.029 | 0.065 | 279 | 3.99 | -1.46 |
| 109 | 0.3936 | 0.3289 | 0.5 | 38.25 | 12.58 | 31.96 | 2.496 | 7.357 | 0.067 | 187 | 0.00 | 2.50 |
| 110+ | 0.4041 | 1.0000 | 2.47 | 25.67 | 25.67 | 63.53 | 2.475 | -- | -- | -- | -- | 2.47 |
